# Successes and challenges of north–south partnerships – key lessons from the African/Asian Regional Capacity Development projects

**DOI:** 10.3402/gha.v9.30522

**Published:** 2016-10-06

**Authors:** Rosanna Färnman, Vishal Diwan, Merrick Zwarenstein, Salla Atkins, for the ARCADE consortium

**Affiliations:** 1 Department of Public Health Sciences (Global Health/IHCAR), Karolinska Institutet, Stockholm, Sweden; 2 Department of Public Health and Environment, R.D. Gardi Medical College, Ujjain, India; 3 Centre for Studies in Family Medicine, Department of Family Medicine, Schulich School of Medicine and Dentistry, Western University, London, ON, Canada

**Keywords:** institutions, partners, students, north–south collaboration, global health, course development

## Abstract

**Introduction:**

Increasing efforts are being made globally on capacity building. North–south research partnerships have contributed significantly to enhancing the research capacity in low- and middle-income countries (LMICs) over the past few decades; however, a lack of skilled researchers to inform health policy development persists, particularly in LMICs. The EU FP7 funded African/Asian Regional Capacity Development (ARCADE) projects were multi-partner consortia aimed to develop a new generation of highly trained researchers from universities across the globe, focusing on global health-related subjects: health systems and services research and research on social determinants of health. This article aims to outline the successes, challenges and lessons learned from the life course of the projects, focusing on the key outputs and experiences of developing and implementing these two projects together with sub-Saharan African, Asian and European institution partners.

**Design:**

Sixteen participants from 12 partner institutions were interviewed. The data were analysed using thematic content analysis, which resulted in four themes and three sub-categories. These data were complemented by a review of project reports.

**Results:**

The results indicated that the ARCADE projects have been successful in developing and delivering courses, and have reached over 920 postgraduate students. Some partners thought the north–south and south–south partnerships that evolved during the project were the main achievement. However, others found there to be a ‘north–south divide’ in certain aspects. Challenges included technical constraints and quality assurance. Additionally, adapting new teaching and learning methods into current university systems was challenging, combined with not being able to award students with credits for their degrees.

**Conclusion:**

The ARCADE projects were introduced as an innovative and ambitious project idea, although not designed appropriately for all partner institutions. Some challenges were underestimated from the beginning, and for such future projects, a more structured approach needs to be adopted. ARCADE partners learned that integrating courses into current university systems and awarding students credits are essential.

## Introduction

Increasing evidence suggests that national capacity for high-quality health research translates into improved population health status (1), strengthening the case for undertaking global health research in low- and middle-income countries (LMICs) (2) and building the capacity to undertake this research within these countries (3–5). Novel research could translate into evidence-based policies (6), which are needed to achieve health agendas, such as the sustainable development goals. North–south collaboration can be very effective to develop sustainable research capacity in LMICs (7). Increasing efforts on global capacity building and north–south research partnerships are being made and have contributed significantly to enhancing the research capacity in the LMICs over the past few decades (8). However, there persists a lack of skilled researchers to inform health policy development, particularly in LMICs (9, 10). This skilled pool of researchers is integral to meeting the health needs of LMICs, many of which are lagging behind in health goals (11), but it is often depleted by a ‘brain drain’ (3), as some of the most talented young health researchers pursue careers elsewhere.

Building capacity at both individual and institutional levels to conduct high-quality research is key to increasing the research output in LMICs (9, 10, 12). The process of capacity building, however, needs to be a concerted effort, structured around relevant research projects, disseminating findings to a large and diverse audience and focusing on the application of those findings (4). Increasing efforts have been made in building capacity in recent years, particularly with large consortium-based capacity building projects, for example CHEPSAA and INTREC (13, 14). The African/Asian Regional Capacity Development (ARCADE) for Health Systems and Services Research (HSSR) and Research on Social Determinants of Health (RSDH) (15) are two such large consortia-based projects that were implemented to build capacity in research in LMICs, in order to ultimately contribute towards health policies. The projects were launched in 2011 with funding from the European Commission FP7 Programme. ARCADE HSSR was established with African and European partners, and ARCADE RSDH was established with Asian and European partners. The aim was to give students and institutions in LMIC easy access to research education online. Both ARCADE projects included four interlinked components (15):

1. Development of online courses on global health topics, in-person and online training sessions, as well as digital training and reading materials
2. Institutional capacity development on communication, research dissemination and grants management
3. New north–south and south–south network development for training and research, including proposal writing
4. Delivery of courses on global health thematic topics, methods and analysis

The ARCADE projects aimed to help build capacity for a new generation of researchers from universities across the globe to gain research training. All project objectives were intended to be contributed to by most partners, working together on courses, institutional capacity and communications. This article seeks to understand participant perceptions of the successes, challenges and lessons learned during the course of the 4-year project, through collecting and analysing senior participant perceptions of the successes, failures and lessons of the ARCADE projects in contributing to research capacity development and to examine project outcomes.

## Methods

### The consortia

The ARCADE projects used innovative educational technologies to strengthen health research across Africa and Asia. The projects focused on postgraduate, doctoral and postdoctoral training. The partner institutions developed cutting-edge online courses, blended learning modules and joint programmes that enabled training of researchers in LMICs who might not otherwise have access to such material. Additionally, the ARCADE projects worked at an institutional level to strengthen education services, financial and administrative research management, research uptake capacity and network building.

Both projects were coordinated by Karolinska Institutet in Sweden, and in total 16 partners across Europe, Africa and Asia were involved (Table 1). The European Commission's 7th Framework Programme funded the 4-year projects (2011–2015).

**Table 1:** ARCADE partners

Institution and department	Country	ARCADE project
Karolinska Institutet, Global Health	Sweden	HSSR, RSDH
Makerere University, College of Health Sciences	Uganda	HSSR
Stellenbosch University, Faculty of Health Sciences	South Africa	HSSR, RSDH
Institute of Development Studies, University of Sussex	UK	HSSR, RSDH
Muhimbili University of Health and Allied Sciences	Tanzania	HSSR
University of Malawi, College of Medicine	Malawi	HSSR
Tongji Medical College of HUST	China	RSDH
Public Health Foundation of India	India	RSDH
Indian Institute of Health Management Research	India	RSDH
Beijing Normal University, School of Social Development and Public Policy	China	RSDH
University of Tampere	Finland	RSDH
Zhejiang University	China	RSDH
Ujjain Charitable Trust Hospital & Research Centre	India	RSDH
Sultan Qaboos University	Oman	RSDH
Hanoi Medical University	Vietnam	RSDH
St. John's National Academy of Health Sciences, CBCI	India	RSDH

Each project had approximately 2–3 annual project meetings in different partner countries. Partners with less funding, also referred to as ‘smaller partners’, were those who were less involved in course preparation and course delivery. The funding division, involvement and engagement of partners were discussed during annual project meetings.

### Approach

This was a qualitative interview study of participants combined with descriptive analysis of project information.

### Participants

All 16 ARCADE partner institutions were approached for interviews. In total, 16 participants from 12 institutions participated – nine men and seven women. Seven of participants were from the RSDH consortium, three were from the HSSR consortium and two institutions were partners in both. All participants had been involved in the project long enough to have a good sense of some of the challenges and successes from the start of the projects. Most participants were project managers and principal investigators, and some had taken leadership roles in specific modules of the project.

### Data collection

The first author (RF) conducted 12 semi-structured interviews in English, through Skype. The interviews were guided by 11 open-ended questions, focusing on experiences, challenges, partnerships, impact and sustainability. Each interview lasted approximately 20–40 min. The interviews were recorded using QuickTime Player and transcribed verbatim. The names and institutions of respondents were concealed. To complement these data, project reports were examined for description of project outputs.

### Analysis

The data were analysed using thematic content analysis (16). It focused on both the manifest (apparent) and latent (ulterior) content of the text. Initially, transcripts were read and re-read to familiarise with the data. Following that, the transcripts were coded and these codes then developed into ‘meaning units’ and finally themes. Members of the coordinating organisation's project management team (RF and SA) analysed the data. RF coded the data, and SA validated the codes and meaning units. Disagreements in the coding and analysis were resolved by discussion.

The data resulted in four themes. These themes are combined with project outcomes as identified from project reports. Each respondent is identified in the text by their role in the project, country category, as well as the funding they had available from the project (high, medium and low funding), which also reflected the contribution that was expected from them.

### Ethics

Each interviewee gave written consent to being interviewed and to be recorded on Skype. The participants were acting purely in their professional capacity as principal investigators during their interviews, thus no additional ethical clearance was needed.

## Results

Descriptive data obtained from project reports indicated that during the 4 years of implementation, across both projects, there were 25 blended courses (courses consisting of an online component and real-time interaction with peers and lecturers) and 30 self-learning modules (course materials that could be used without interaction with peers and lecturers) developed. ARCADE RSDH, the larger consortium, developed the balance of the modules, while 11 and 14 blended learning courses were developed by ARCADE HSSR and ARCADE RSDH, respectively. Most courses were developed cross-institutionally, as courses were intended to be used on an open-access principle. Therefore, some courses were hosted either on institutional platforms and others made public on the ARCADE projects’ joint online learning platform (www.courses.arcade-project.org/), which currently hosts 20 courses or modules. In total, these courses reached over 900 postgraduate students, of whom more than 270 took part in courses in HSSR and almost 650 in RSDH. Approximately 55% of all participating students were female. In addition to student training, the consortia wrote 60 research funding proposals, of which 50 included support for PhD funding. Of these, approximately 20% were funded. Four research management training courses for university research administration and financing personnel were held and communication capacity was also built across institutions through workshops and online webinars. In addition, Karolinska Institutet and Makerere University (which already had their own joint doctoral degree programme) each signed and implemented a joint degree programme with the University of Stellenbosch.

On both master and PhD level, ARCADE courses reached more female students than male students, as expected and planned for when the projects were initiated. Successfully, some courses, such as ‘Behavioural change communication’ attracted a high number of female students, whereas methods courses attracted a close to even number between males and females. The level of dropout or failure during these courses was very low (at most two students per course).

In addition to the courses developed and implemented, the projects also conducted several workshops in grants management and enhanced communication capacity, and involved junior researchers in proposal writing and mentoring.

While these were the quantifiable outputs of both ARCADE projects, much of the experiences in capacity building are not measurable: these may focus on relationship dynamics, work and the learning experienced by the participants involved. Below, we present the results of a qualitative analysis, focusing on what were the key experiences and lessons learned from the ARCADE collaboration for the researchers involved in the study. We identified four main themes and three sub-categories in the analysis, which are described in more detail below.

### EU projects: lessons in bureaucracy

Participants reported that working within an EU project was an institutional accomplishment for several institutions. Those who had not taken part in such projects before learned how to work in a global environment, with multiple partners. In particular, two of the smaller institutions appreciated the guidance provided by larger partners and especially their willingness to share from their previous experiences of working in EU projects. However, despite the sense of accomplishment, EU projects were considered bureaucratic, excessively restrictive and very time-consuming in terms of reporting.

This burdensome administration of the projects towards the EU was more evident during changes in the coordination team at KI, as project managers and research assistants changed, as work package leaders changed and when new partners joined. Participants considered these as stressful and challenging for project implementation, impacting communication across projects negatively. Some partners also felt that change in human resources at the coordinator affected consortium functioning as a whole.

The responsibilities have changed many times in the coordinating institutions, this may have had some delay in the deliverables or communication with the partners, different people … taking over. (Principal Investigator, Middle Income Country, medium funding)

The formal nature of the projects also meant that arrangement for working together needed to be structured and tightly scheduled. This was complicated by the fact that long distances and substantial time differences in such a global project were serious barriers to communication. Despite careful planning, poor Internet connections and partners who had problems in attending meetings due to time differences had a negative influence on project work.

### E-learning as a new concept bringing new challenges

The EU projects brought in resources and opportunities for high-level postgraduate training in the partner institutions in southern Africa and Asia, including innovative teaching and self-learning approaches. According to several partners, the ARCADE projects were ambitious and timely concepts that brought e-learning to the agenda for many institutions that had never used such tools for teaching and learning before. Many of those involved from these regions reported a change in mindset from negative to positive with regard to the power and effectiveness of e-learning and blended methods as they were exposed to the idea. Some partners even started spin-off projects inspired by ARCADE. These included mobile apps used to market the projects to students and inform them of the courses to be run in their institutions.

Participants saw the project as creating a platform for cross-country and cross-institutional teaching and learning, and for creating long-term relationships with institutions with which they were not previously familiar. This was seen as providing opportunities for smaller institutions, with less resources and infrastructure for teaching, to introduce e-learning to their students in ways that had not been previously possible. There was also the opportunity to create new or revised courses, as informed by the needs assessment conducted at the beginning of the project, using an e-learning approach.

Distance learning is the future, which we are underestimating in our countries. Without ARCADE we would possibly not have started with e-learning or blended learning until much later, if at all. (Principal Investigator, Middle Income Country, medium funding)

As the e-learning focus was new to most participants, the approach taken to the work was primarily ‘learning by doing’. When the projects started in 2011, there were far fewer online learning resource platforms than at present, and thus, few of the partners had prior experience with such platforms. Though ARCADE is now one programme among many, many participants reported that there are positive differences between the ARCADE platform and other online sources for teaching and learning:

The global impact of [ARCADE] is that it is not made by top-notch researchers in Western world, trying to educate people in developing countries. It's actually these universities from developing countries, professors from developing countries, customizing and making courses that are tailored for their students. Giving the experiences of the international professors from UK or Sweden, who are involved, so giving their experiences but the courses were customized and tailored to the needs of these. That makes a difference from other courses that are made online from other universities globally. (Project Manager, High Income Country, low funding)

The project deliverables involved developing and delivering courses along with other capacity building activities. However, many participants reported that new relationships developed during the project implementation were the most important outcomes. They reported having benefited substantially from visits to other institutions, discovering how they made effective use of e-learning and, in particular, blended learning approaches.

#### Underestimating technical challenges

Despite these beneficial impacts, the approach the consortia took to the project could have been improved. At the beginning of the ARCADE, partners did not have a clear understanding as to how course implementation and management would work in practice. Some partners found that basic technical and logistical issues were initially not sufficiently acknowledged:

We were probably underestimating the technical, logistical and institutional difficulties of bringing the whole thing together at the start … we didn't take [these challenges] on-board efficiently at the start. Simple things like bandwidth and technical expertise to get these things online … It would have been advantageous from the beginning of ARCADE to just focus on that issue of quality and to maybe limit our ambitions on a limited number of very high quality outputs and show what you can do if you just focus on the quality side. (Project Officer, High Income Country, high funding)

These technical challenges impacted on the development and online management of courses. The projects were intended to be available online globally for PhD students, but ironically, the southern partners had less access to these resources. They struggled with insufficient bandwidth in delivering courses, and for the same reason even had difficulties participating in project meetings via Skype.

Definitely there were some challenges. It's a nice global project spread across three continents, but a challenge was bandwidth. Even if we can have Skype meetings, there were various technical issues. Distance is still a big issue … [Also], the communication strategy could have been more intensive, so we could have done more (Project Officer, Middle Income Country, low funding)

Though these technological challenges persisted, partners still wished for increased opportunities for participation over the Internet. In particular, they proposed that seminars and grants management workshops should be covered by video link, for others to watch. Here, the technological challenges met with a lack of human resources, as there was no one to implement these recordings. Partners considered these missed opportunities on issues that related to a major component of the project, felt that there could have been an intensive communication strategy and that more could have been done.

Another important point about the workshops, as part of the ARCADE, the workshops that we organized were very important, innovative and relevant topics but the majority of them were onsite and at one time. If these workshops in the future can be converted into online-mode, or webinars, or even if these workshops are onsite workshop, they can be developed or converted into e-learning courses for sustainability point of view… (Project Manager, Middle Income Country, medium funding)

### Adapting the project to current university systems

ARCADE introduced a new way of learning that challenged the traditional teaching methods in several institutions. This was particularly evident in some Asian institutions, where the learning culture among students proved to be less open to new methods, a problem that possibly needed more time to overcome or more evidence of the opportunities that e-learning could bring. However, some partners argued that the 4-year duration of the ARCADE projects should have been sufficient to bring courses in line with institutional accreditation and thus bring students to the courses:

Four years is a sufficient time to market the ARCADE product, so we could have done more. But there were challenges because we were not sure about the degree, certificate, and accreditation. This is what the PhD and other students’ want. (Principal Investigator, Middle Income Country, low funding)

Many partners also found that ARCADE, as an external project, was difficult to fit into existing university systems:

In some universities there were actually university statutes that were rather difficult to move into these new areas. Requirements that students were physically in contact with people for a minimum period of time, which obviously made great sense for traditional courses, but not for some of the courses that people wanted to put on. (Project officer, High Income Country, high funding)

This new mode of teaching and learning was in many of the partner institutions as new to teachers as it was to students. To some extent, the challenge of embedding ARCADE courses in curricula remained the major problem for teachers even when students had accepted their value. A number of the partners reported that, once this acceptance had been attained, students were perfectly willing to take a more independent approach to their studies:

The students have learnt what is the meaning of learning courses. I encourage my students to go on the webpage to learn more for themselves and to have self-learning. The total hours for the course was 32 hours but now I want them to have more self-learning and I encourage them to give more presentations and the student may be more active. (Principal Investigator, Middle Income Country, low funding)

#### Efforts to attract students

Partners also reported that the language of instruction impacted on course development, especially in ARCADE RSDH. In China, most courses had to be translated from English to Chinese, in order to make the courses available for all students. Chinese partners also struggled with national Internet restrictions and this could affect the possibility to download material from the ARCADE platform. The Chinese partners were also among those who found it difficult to make students interested in this new form of learning online:

We are more open to face-to-face courses. Not many students want to learn from courses online. Many of the websites are also forbidden in China. Face-to-face is also the traditional way to learn, and not so many students accept this online way … two years ago I tried to learn a MOOC course, but I couldn't download the video. Maybe in Europe or in the US it is easier to download. Maybe more students will participate in the future. Most of the online courses are actually better that those face-to-face. (Principal Investigator, Middle Income Country, low funding)

Accreditation was another great challenge for institutions when introducing ARCADE courses to their students and universities. Some students and teachers were happy to take part in courses just to gain more knowledge within an area; however, most students were understandably only open to taking courses if they are awarded credits for their degree. ARCADE struggled in this area during the projects, except where Karolinska Institute and Makerere University partnered and created joint degree programmes. Though teaching resources were developed, they were not necessarily taken on by university curricula. Also, in Chinese universities, students could only get 2–3 credits for courses done outside the home university, thus reducing the attraction of even European Credit Transfer System (ECTS) accredited courses.

The projects also encouraged students to engage in study visits and workshops, within countries and between them. Some students met with other partner institutions’ students and developed proposals together, a rare opportunity:

They really learned something new, about how to manage grants and how to write budgets, manage the financial reports to the donor agents, how to manage communication with the donor partners and basically how to get legal appraisal of the projects or financial appraisals of the project. (Project Manager, Middle Income Country, medium funding)

### Challenges and successes in collaboration

One of the main benefits of the ARCADE projects was the opportunity to establish new or improved collaborations between institutions. According to the institutions that were part of both ARCADE HSSR and ARCADE RSDH, the African network had a well-balanced number of partners, and several of them already knew each other. In contrast, some partners in ARCADE RSDH indicated that the Asian consortium did not function as well as they had hoped. Participants thought this could have been because many of the partners had no previous interaction and had different views as to the overall purpose of the project. Some institutions in ARCADE RSDH found it difficult to reach out and establish new collaborations with other southern partners. This was not perceived as an issue in the ARCADE HSSR consortium. In particular, as new partners joined after the initial phase of the Asian project, it was perceived as difficult to clearly define their role within the consortium and to hold joint seminars with participants that had not previously met face-to-face.

The [HSSR] network itself was also much smaller … people know each other and talk to each other and maintain a connection; whereas the Asian network is more dispersed, there were more partners and most partners had existing collaborations with separate European partners …. There were stronger bilateral links rather than having partnerships between each other, between Asian partners. I think these tendencies maintained, I guess it also has to do with their universities and what is valued. They have this value of having connections to Western partners, rather than having local networks… (Research assistant, High Income Country, high funding)

For some partners, it was clear that over time successful collaborations would probably emerge, as ARCADE successfully introduced partners to each other that had not previously had any communications. Some regional southern collaboration grew during the ARCADE project, and participants believed these would continue post-funding. Probably the most successful form of collaboration was based around joint preparation of research proposals, and in that way exploring each other's areas of expertise.

I think the project has injected some momentum for collaboration. Imagine four years ago, I didn't know anything about the Indian institute, Chinese institutes … without the ARCADE this hadn't started (sic) … this is just the beginning and I hope it will continue. (Project Manager, High Income Country, low funding)

#### The ‘north–south divide’

Others thought that communication had failed in the Asian consortium and, because of this, there was a high risk of smaller partners being left behind in terms of benefits from the project. Accordingly, partners with less funding (almost entirely LMIC partners) confirmed that they felt as though they had less influence in decisions, but this was not surprising to them, as it was expected from large consortia. The European partners, most with larger funding, found that some of the African and Asian institutions took leading roles in the consortium, something that was often sought in such collaborations. However, the consortium struggled with an emphasis on north–south partnerships above south–south partnerships, despite directly aiming to build more southern networks.:

It appeared to be as easy for [African partners to link with each other as it was to link with Northern institutions], but in fact the energy was not as great. Its seems as though South institutions are still looking North for main partnerships, possibly because they have a perception that North partnerships bring money with them, and this one did, because of the ARCADE funding … but most likely it is because organisations in the South are so busy that they know very little about other Southern institutes. (Senior Lecturer, High Income Country, high funding)

The southern partners engaged in the project were well known, and extremely busy, which contributed towards lack of network building. Partners also worked more easily towards reinforcing existing partnerships, which were mainly north–south. In addition, when the project was managed in the north, taking leadership in the south is challenging. However, many of the participants also discussed how collaboration could be changed in future, with one emphasising the need for a tailored approach for each partner institute:

Personally, what I learnt when working through [a work package] was that some of the partners required one-to-one support and others were very happy to get on with what they were delivering. The approach from individual HSSR and RSDH partners was very different in terms of developing those learning modules. In the future I would probably tailor my approach to work with the partners differently … I did some one-to-one work with [a partner] in China and I think both of us benefitted from that … I would also try to do more group things to keep people on track more. (Project Manager, High Income Country, high funding)

## Discussion

This article has drawn together the successes, challenges and lessons learned from the two EU FP7 projects, ARCADE HSSR and ARCADE RSDH. Over four years of funding, the quantitative outcomes by the two consortia indicate a considerable degree of success with strong prospects of sustainability, given that several of these online courses were internalized in university curricula. The issues that were focused on in both projects, HSSR and RSDH, are core competencies in global health (17). Overall, the projects have significantly advanced the overall aim of research capacity building in these partner institutions, but more could be done in the future. Below we outline some of the issues that emerged during the projects.

Cross-cultural, cross-institutional and multidisciplinary north–south partnerships, such as the ARCADEs, can be of high value in building sustainable capacity (7, 8), but they need to be designed appropriately. The ARCADE projects could be seen as a prototype for what could have been an easily accessible and effective model of reaching students globally with freely available courses as well as increasing north–south partnerships. However, four years is a short time to establish sustainable new collaborations and projects. In ARCADE HSSR, where project participants were few and most had previously undertaken joint initiatives, outcomes were easier to achieve than in the larger ARCADE RSDH where some partners were new to each other. EU projects bring prestige to participants, but can also seem burdensome, especially to southern partners, in terms of management and reporting requirements. This can hamper substantive project activity, as institutions, particularly those with less funding, can become more concerned reporting and deliverables than developing and implementing courses and related activities. The ARCADE Open Course Repository, the website where most courses are mounted, will be maintained for at least another two years. However, more time will be needed for the project outputs to be considered fully sustainable. One way to ensure sustainability would have been to engage more policy makers (18) in capacity building efforts. This was one ARCADE project intention that was not fully realised, possibly because the magnitude of this task was underestimated. Future capacity building projects should give this aspect attention.

A major challenge to the ARCADE work was that the project focused on developing and delivering blended courses at universities. It was, however, a project perceived by senior health researchers in those universities to be on a topic, e-learning, that was tangential to their primary areas of interest. Research indicates that in order for educators to act on a reform, they need to be given an opportunity to construct an understanding of the reform (19). Shifting into blended learning is a curricular change (20), and it can be expected that in order for the process to take off, and for lecturers and institutions to adapt, considerable time is needed. Blended learning needs to be aligned with institutional, faculty and student goals (21) in order to be effective. Accordingly, much work was needed in promoting and marketing the project concept to universities, matching the courses to university needs and finally attracting students to courses where they could not see the immediate benefit in terms of gaining credits. The result of this possible mismatch was, for example in ARCADE RSDH, an increased interest in courses delivered from the northern institutions, which students perceived as having a greater depth of expertise than those in their own regional neighbours (W. Yan, personal communication, 2013).

Besides the quantitative outcomes in terms of materials developed and students reached by courses, there are many intangible benefits from the ARCADE projects. Staff from the various institutions learned from each other, met each other and created interpersonal relationships that will outlast the funding cycle. This can have positive effects on research in the LMICs involved, as international collaboration may encourage best practice, promote the sharing of ideas and contribute towards evidence-based policies (22). Staff networked by writing proposals together, learning each other's strengths, and thus created working relationships, both between north–south and south–south, that can contribute towards long-term capacity building. This was a key outcome of the ARCADE projects – international collaboration has been positively linked to a scientist's research output, at least in biomedicine (23), and informal communication such as much of ARCADE's work is important for this collaboration (24). The creation of sustainable southern partnerships was an important goal in ARCADE; but at least in RSDH, contrary to existing research (24), collaborations were not more successful with partners in close proximity when compared with partners further away. However, after the project all institutions are now more familiar with each other and their respective areas of expertise. This familiarity built through ARCADE may in future turn into research collaborations and build stronger linkages between the partners.

As noted in other ARCADE-related publications, lecturers (25), course creation and implementation, (26) as well as students’ experiences of the courses (27), technological challenges were a key challenge to both managing the project and creating the key outputs, also from the perspective of project managers and principal investigators. The technology used for blended learning, which was key to project implementation, is progressing at a fast rate. It can be expected that in future, blended learning will be easier to implement and global collaborations in projects is also easier.

Our study indicates that embarking on capacity building in the south, though it is a key need for many institutions (28), is not a simple process. Capacity building requires a complex set of activities at the level of institution, department and student (4). The ARCADE projects brought together capacity building activities that involved course development, mentoring (29), grant office capacity building and communication capacity building (15). From our four years of experience, we can draw several key lessons (see Box 1) for others embarking on such projects.

Box 1 Recommendations for future capacity building projects

Key lessons from the ARCADE projects

1. Structured approach to communications and coordination from the beginning emphasising equality among partners.
2. Engage skilled IT personnel and e-learning support.
3. Hire dedicated staff for the project at each partner institution
4. Acknowledge institutional constraints and areas of expertise when planning project activities.
5. Reward students who take part in courses, and integrate courses into existing university systems.
6. Focus coordination on facilitating south-south partnerships.
7. Institute quality assurance of courses – focusing on both content and technical quality.
8. Involve policy makers to gain support for project implementation.

## Conclusions

During the four years of project duration, the ARCADEs succeeded in developing content online and in face-to-face courses that support research capacity building, but importantly also built relationships between northern and southern partners, as well as among southern partners. The project implementation was challenged by problems in technology, especially bandwidth and software deficiencies, availability of knowledgeable technical staff and attracting students’ interest in courses. The relationships built during the project, however, along with the courses developed, have the potential to sustain capacity building efforts in future.

## Paper context

The ARCADE projects involved 16 partners globally in building research capacity. The experience of the consortia was positive, but technical, human resource and institutional challenges slowed progress during the project. The project created both tangible outputs in terms of courses and proposals produced and important intangible outputs in relationships made across institutions and countries. Such large networked projects have potential for future research capacity development. We present recommendations for future projects.
